# National and State Cost Savings Associated With Prohibiting Smoking in Subsidized and Public Housing in the United States

**DOI:** 10.5888/pcd11.140222

**Published:** 2014-10-02

**Authors:** Brian A. King, Richard M. Peck, Stephen D. Babb

**Affiliations:** Author Affiliations: Richard M. Peck, University of Illinois at Chicago, Chicago, Illinois; Stephen D. Babb, Centers for Disease Control and Prevention, Atlanta, Georgia.

## Abstract

**Introduction:**

Despite progress in implementing smoke-free laws in indoor public places and workplaces, millions of Americans remain exposed to secondhand smoke at home. The nation’s 80 million multiunit housing residents, including the nearly 7 million who live in subsidized or public housing, are especially susceptible to secondhand smoke infiltration between units.

**Methods:**

We calculated national and state costs that could have been averted in 2012 if smoking were prohibited in all US subsidized housing, including public housing: 1) secondhand smoke-related direct health care, 2) renovation of smoking-permitted units; and 3) smoking-attributable fires. Annual cost savings were calculated by using residency estimates from the Department of Housing and Urban Development and cost data reported elsewhere. Data were adjusted for inflation and variations in state costs. National and state estimates (excluding Alaska and the District of Columbia) were calculated by cost type.

**Results:**

Prohibiting smoking in subsidized housing would yield annual cost savings of $496.82 million (range, $258.96–$843.50 million), including $310.48 million ($154.14–$552.34 million) in secondhand smoke-related health care, $133.77 million ($75.24–$209.01 million) in renovation expenses, and $52.57 million ($29.57–$82.15 million) in smoking-attributable fire losses. By state, cost savings ranged from $0.58 million ($0.31–$0.94 million) in Wyoming to $124.68 million ($63.45–$216.71 million) in New York. Prohibiting smoking in public housing alone would yield cost savings of $152.91 million ($79.81–$259.28 million); by state, total cost savings ranged from $0.13 million ($0.07–$0.22 million) in Wyoming to $57.77 million ($29.41–$100.36 million) in New York.

**Conclusion:**

Prohibiting smoking in all US subsidized housing, including public housing, would protect health and could generate substantial societal cost savings.

## Introduction

Exposure to secondhand smoke from burning tobacco products causes disease and premature death among nonsmokers (1). Each year, secondhand smoke exposure is responsible for an estimated 7,330 deaths from lung cancer and more than 33,950 deaths from heart disease among US adult nonsmokers (2). Additionally, lost productivity resulting from exposure to secondhand smoke is estimated to cost the United States approximately $5.6 billion annually (2). The US Surgeon General concluded that no risk-free level of secondhand smoke exists and that eliminating smoking in indoor spaces is the only effective way to fully protect nonsmokers from the adverse effects of secondhand smoke exposure (1).

In the United States, considerable progress has been made toward increasing the number of statewide comprehensive smoke-free policies that prohibit tobacco smoking in all indoor areas of public places and worksites, including restaurants and bars. As of January 2014, 26 states and the District of Columbia had enacted comprehensive smoke-free policies (3). Such policies reduce secondhand smoke exposure and the incidence of certain adverse health events among nonsmoking hospitality workers and the general public (1,4). However, these policies do not eliminate secondhand smoke exposure from all environments. Private settings such as homes remain a major source of secondhand smoke exposure for many people, especially children (1). Nearly all nonsmokers who live with someone who smokes inside their home are exposed to secondhand smoke (5).

Multiunit housing residents are particularly susceptible to involuntary secondhand smoke exposure in the home. Environmental studies indicate that secondhand smoke constituents can infiltrate units where no smoking occurs (eg, units whose residents have adopted smoke-free home rules) from units and shared areas where smoking is permitted (6,7). Additionally, research indicates that many of the nearly 80 million Americans who live in multiunit housing experience secondhand smoke infiltration in their living unit that originated from elsewhere in or around their building (8). Nearly 7 million US multiunit housing residents live in government subsidized housing, including approximately 2 million in public housing either owned or operated by a government housing authority (9). The potential for secondhand smoke exposure in public or subsidized housing is of particular concern because a large proportion of these units are occupied by people who are particularly sensitive to secondhand smoke, including children (45%), the elderly (41%), and the disabled (25%) (9).

In addition to increasing the health burden and health care costs, exposure to secondhand smoke in multiunit housing can also lead to considerable financial costs for renovation of units in which smoking has occurred and smoking-attributable fires (10,11). Research suggests that prohibiting smoking in all US subsidized housing nationwide would yield cost savings of approximately $521 million per year, including $154 million annually for public housing (10). However, the cost savings that could be achieved by prohibiting smoking in these settings at the state level is uncertain. The objective of our study was to assess the state-specific costs that could have been averted if smoking were prohibited in US subsidized housing in 2012, including those from secondhand smoke-related direct health care, renovation of units where smoking has occurred, and smoking-attributable fires. Estimated cost savings were calculated for subsidized housing overall (including public housing) and for public housing alone.

## Methods

Cost savings estimates were calculated by using the approach of King and colleagues (10), which we modified to account for inflation, state variations in health care and living costs, and state differences in key indicators such as subsidized and public housing residency, tenant turnover rates, Medicaid enrollment, and smoke-free home rule prevalence. Costs that could be averted by prohibiting smoking in subsidized housing were calculated for 3 factors: 1) secondhand smoke-related health care; 2) renovation of smoking-permitted units; and 3) smoking-attributable fires. The approach used to estimate cost savings across each of these 3 factors is outlined in the accompanying diagram using New York as an example; in brief, annual cost savings were calculated using subsidized housing residency estimates, and data were adjusted for inflation and variations in other costs across states (Figure).

**Figure:**
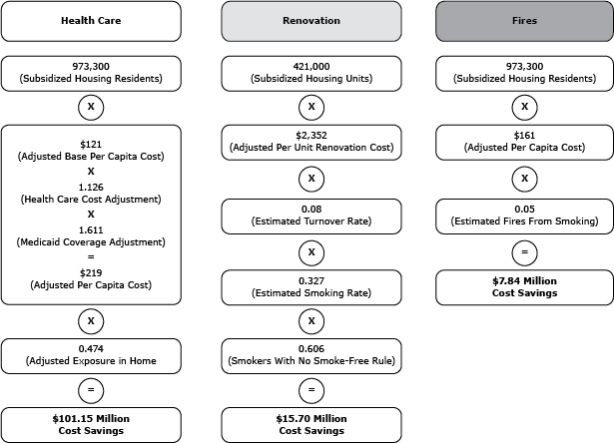
Example of calculations used to estimate cost savings associated with prohibiting smoking in subsidized housing, New York State.

### Health care costs

Expenditures for health care related to secondhand smoke exposure were based on published cost estimates from a study conducted among nonsmoking Minnesota residents. The estimates were derived from claims data from the state’s largest health insurer (12). Because Minnesota’s overall smoking prevalence is approximately half that of subsidized housing residents (3,10), the annual per capita savings reported for Minnesota ($44) were adjusted to $85 using the smoking prevalence for each of these populations (32.7/16.8 multiplied by $44). This value was then adjusted to 2012 dollars ($91) using the consumer price index (13). To account for differences in living costs across states, $85 was multiplied by a price deflator, which was calculated by dividing each state’s 2012 cost of living index by Minnesota’s cost of living index (13). This value was multiplied by the number of subsidized and public housing residents in each state (9) and adjusted for the approximate percentage of total secondhand smoke exposure occurring in the home (58.4%) (14), state variation in the prevalence of smoke-free home rules (15), per capita health care expenditures (16), and the proportion of the population enrolled in Medicaid (17). Alaska and the District of Columbia were excluded because of lack of data (9). The final value for each state is the estimated health care cost savings for all nonsmoking residents of subsidized and public housing.

### Renovation costs

The cost of renovating units where smoking occurred was calculated by multiplying the state’s number of occupied subsidized and public housing units (excluding Alaska and District of Columbia) by the average annual turnover rate for subsidized and public housing in each state (9), the estimated prevalence of adult smoking in subsidized housing (32.7%) (10), and an adjustment for the percentage of smokers with smoke-free home rules in each state (15). The resulting value for each state was then multiplied by the state’s estimate of the excess cost of renovating a single unit that permits smoking. State estimates of excess renovation costs were obtained by using the average ($1,674) of a previously published range ($770–$2,170) from the Smoke-Free Housing Coalition of Maine (18). This excess renovation estimate was adjusted to 2012 dollars by using the consumer price index ($1,906) (13), and a price deflator was applied to account for variations in living costs across states: the deflator was calculated by dividing each state’s 2012 cost of living index by Maine’s cost of living index (13).

### Smoking-attributable fire costs

The cost associated with smoking-attributable fires was calculated by multiplying the state’s number of subsidized and public housing residents by National Fire Protection Association estimates of the annual per capita loss (including property damage, deaths, and injuries) from all US fires ($151) (19), which was adjusted to 2012 dollars using the consumer price index ($161), and by the percentage of fires caused by cigarettes (5.0%) (20). These national estimates were applied to each state because of the lack of state-specific data for this indicator.

### Sensitivity analysis

A sensitivity analysis was performed to develop a range for each cost savings estimate by using an approach described by King and colleagues (10). For health care costs, we used the range of per-capita secondhand smoke expenditures from Waters and colleagues ($56–$121) (12), which was adjusted to 2012 dollars by using the consumer price index ($60–$129) (13); the assumed average percentage of time spent in public housing was 43.8% to 73.0% (14). For renovation costs, it was assumed that per-unit costs and turnover rates were 75% to 125% of baseline figures for each state. For smoking-attributable fire costs, it was assumed that per-capita losses from all fires and the proportion of smoking-related fires were 75% to 125% of baseline figures for each state. Variations in state living costs were accounted for in the sensitivity analysis by using the same approach that was used to calculate the baseline figures.

## Results

### All subsidized housing

Prohibiting smoking in all US subsidized housing would yield estimated annual cost savings of $496.82 million (range, $258.96–$843.50 million), including $310.48 million ($154.14–$552.34 million) in secondhand smoke-related health care, $133.77 million ($75.24–$209.01 million) in renovation of smoking-permitted units, and $52.57 million ($29.57–$82.15 million) in smoking-attributable fire losses (Table 1).

**Table 1:** Estimated Annual Cost Savings Associated With Prohibiting Smoking in Subsidized Housing, by Cost Type, United States, 2012

State	Health Care, $ Million (Range)^a^	Renovation, $ Million (Range)^a^	Fires, $ Million (Range)^a^	Total, $ Million (Range)^a^
Alabama	6.09 (3.02–10.84)	5.03 (2.83–7.86)	1.27 (0.71–1.98)	12.39 (6.57–20.67)
Alaska	—b	—b	—b	—b
Arizona	3.54 (1.76–6.30)	0.76 (0.43–1.18)	0.61 (0.34–0.95)	4.90 (2.52–8.43)
Arkansas	2.96 (1.47–5.26)	3.25 (1.83–5.08)	0.59 (0.33–0.92)	6.80 (3.63–11.27)
California	61.08 (30.32–108.66)	5.86 (3.30–9.16)	5.42 (3.05–8.46)	72.36 (36.67–126.28)
Colorado	2.20 (1.09–3.91)	1.62 (0.91–2.53)	0.72 (0.40–1.12)	4.53 (2.40–7.55)
Connecticut	7.88 (3.91–14.02)	1.99 (1.12–3.10)	0.82 (0.46–1.29)	10.69 (5.49–18.41)
Delaware	0.95 (0.47–1.68)	0.25 (0.14–0.39)	0.11 (0.06–0.17)	1.30 (0.67–2.24)
District of Columbia	—b	—b	—b	—b
Florida	15.84 (7.86–28.17)	4.79 (2.69–7.48)	2.62 (1.47–4.10)	23.24 (12.03–39.75)
Georgia	6.69 (3.32–11.91)	3.26 (1.84–5.10)	1.69 (0.95–2.64)	11.64 (6.11–19.64)
Hawaii	2.78 (1.38–4.95)	0.66 (0.37–1.02)	0.32 (0.18–0.50)	3.76 (1.93–6.47)
Idaho	0.45 (0.22–0.80)	0.23 (0.13–0.36)	0.14 (0.08–0.21)	0.82 (0.43–1.38)
Illinois	7.37 (3.66–13.12)	4.53 (2.55–7.07)	1.35 (0.76–2.11)	13.25 (6.97–22.30)
Indiana	3.73 (1.85–6.64)	3.68 (2.07–5.75)	0.91 (0.51–1.42)	8.32 (4.43–13.81)
Iowa	1.66 (0.82–2.95)	1.71 (0.96–2.68)	0.38 (0.21–0.60)	3.75 (2.00–6.23)
Kansas	1.02 (0.50–1.81)	1.54 (0.87–2.40)	0.29 (0.17–0.46)	2.85 (1.54–4.67)
Kentucky	2.68 (1.33–4.77)	3.82 (2.15–5.96)	0.63 (0.36–0.99)	7.13 (3.83–11.72)
Louisiana	9.27 (4.60–16.48)	3.83 (2.16–5.99)	1.33 (0.75–2.08)	14.43 (7.50–24.55)
Maine	3.07 (1.53–5.47)	0.89 (0.50–1.38)	0.27 (0.15–0.42)	4.23 (2.17–7.27)
Maryland	4.57 (2.27–8.14)	1.66 (0.94–2.60)	0.71 (0.40–1.11)	6.95 (3.61–11.85)
Massachusetts	18.20 (9.03–32.37)	4.31 (2.42–6.73)	1.51 (0.85–2.37)	24.02 (12.31–41.47)
Michigan	7.07 (3.51–12.58)	4.37 (2.46–6.83)	1.32 (0.74–2.06)	12.77 (6.71–21.48)
Minnesota	4.14 (2.05–7.36)	2.24 (1.26–3.50)	0.75 (0.42–1.17)	7.13 (3.74–12.03)
Mississippi	4.46 (2.21–7.93)	1.95 (1.10–3.04)	0.75 (0.42–1.17)	7.16 (3.73–12.15)
Missouri	4.36 (2.17–7.76)	4.03 (2.26–6.29)	1.03 (0.58–1.62)	9.42 (5.01–15.66)
Montana	0.44 (0.22–0.79)	0.47 (0.26–0.73)	0.13 (0.07–0.20)	1.04 (0.55–1.72)
Nebraska	0.97 (0.48–1.73)	0.91 (0.51–1.42)	0.26 (0.14–0.40)	2.14 (1.14–3.55)
Nevada	1.07 (0.53–1.90)	0.52 (0.29–0.81)	0.36 (0.20–0.56	1.94 (1.02–3.27)
New Hampshire	0.99 (0.49–1.77)	0.68 (0.38–1.06)	0.20 (0.11–0.31)	1.87 (0.99–3.14)
New Jersey	9.19 (4.56–16.34)	4.71 (2.65–7.37)	1.85 (1.04–2.89)	15.75 (8.25–26.6)
New Mexico	2.08 (1.03–3.70)	0.58 (0.32–0.90)	0.29 (0.16–0.46)	2.95 (1.52–5.06)
New York	101.15 (50.22–179.9)	15.70 (8.83–24.53)	7.84 (4.41–12.24)	124.68 (63.45–216.71)
North Carolina	6.98 (3.47–12.42)	5.42 (3.05–8.47)	1.54 (0.86–2.40)	13.94 (7.38–23.29)
North Dakota	0.45 (0.22–0.80)	0.60 (0.34–0.94)	0.12 (0.07–0.19)	1.18 (0.63–1.93)
Ohio	11.34 (5.63–20.18)	7.98 (4.49–12.47)	2.41 (1.36–3.77)	21.73 (11.48–36.41)
Oklahoma	3.10 (1.54–5.51)	3.07 (1.73–4.80)	0.61 (0.35–0.96)	6.78 (3.61–1.93)
Oregon	2.70 (1.34–4.80)	1.07 (0.60–1.68)	0.51 (0.29–0.79)	4.28 (2.23–7.28)
Pennsylvania	9.26 (4.60–16.48)	6.82 (3.84–10.66)	1.63 (0.91–2.54)	17.71 (9.35–29.68)
Rhode Island	2.63 (1.31–4.68)	1.06 (0.60–1.66)	0.31 (0.17–0.48)	4.00 (2.08–6.82)
South Carolina	3.45 (1.71–6.13)	2.82 (1.58–4.40)	0.75 (0.42–1.18)	7.02 (3.72–11.71)
South Dakota	0.50 (0.28–0.89)	0.50 (0.28–0.78)	0.11 (0.06–0.18)	1.11 (0.59–1.84)
Tennessee	6.35 (3.15–11.29)	5.34 (3.00–8.34)	1.26 (0.71–1.96)	12.94 (6.86–21.59)
Texas	15.87 (7.88–28.22)	8.69 (4.89–13.57)	3.71 (2.09–5.79)	28.26 (14.85–47.59)
Utah	0.68 (0.34–1.21)	0.42 (0.24–0.66)	0.25 (0.14–0.38)	1.35 (0.72–2.26)
Vermont	1.46 (0.73–2.60)	0.33 (0.18–0.51)	0.13 (0.07–0.20)	1.92 (0.98–3.31)
Virginia	4.02 (1.99–7.15)	2.51 (1.41–3.93)	1.23 (0.69–1.92)	7.76 (4.10–12.99)
Washington	3.39 (1.68–6.03)	1.08 (0.61–1.69)	0.55 (0.31–0.87)	5.02 (2.60–8.58)
West Virginia	1.75 (0.87–3.12)	2.27 (1.27–3.54)	0.32 (0.18–0.50)	4.34 (2.32–7.16)
Wisconsin	3.97 (1.97–7.06)	1.85 (1.04–2.89)	0.65 (0.36–1.01)	6.47 (3.37–10.96)
Wyoming	0.19 (0.10–0.35)	0.33 (0.19–0.52)	0.05 (0.03–0.08)	0.58 (0.31–0.94)
United States^c^	310.48 (154.14–552.34)	133.77 (75.24–209.01)	52.57 (29.57–82.15)	496.82 (258.96–843.50)

a Estimates by cost type may not equal total because of rounding.

b Estimates not presented because of lack of data on subsidized housing residency.

c Estimates exclude Alaska and the District of Columbia.

By state, total annual cost savings for subsidized housing ranged from $0.58 million ($0.31–$0.94 million) in Wyoming to $124.68 million ($63.45–$216.71 million) in New York (Table 1). By cost-type, state annual cost savings for secondhand smoke-related health care ranged from $0.19 million ($0.10–$0.35 million) in Wyoming to $101.15 million ($50.22–$179.9 million) in New York. State annual cost savings for renovation of smoking-permitted units ranged from $0.23 million ($0.13–$0.36 million) in Idaho to $15.70 million ($8.83–$24.53 million) in New York; and state annual cost savings for smoking-attributable fire losses ranged from $0.05 million ($0.03–$0.08 million) in Wyoming to $7.84 million ($4.41–$12.24 million) in New York.

### Public housing

Prohibiting smoking in public housing alone would yield estimated annual cost savings of $152.91 million ($79.81–$259.28 million), including $94.01 million ($46.67-$167.24 million) in secondhand smoke-related health care, $42.99 million ($24.18–$67.17 million) in renovation of smoking-permitted units, and $15.92 million ($8.95–$24.87 million) in smoking-attributable fire losses (Table 2).

**Table 2:** Estimated Annual Cost Savings Associated With Prohibiting Smoking in Public Housing, by Cost Type, United States, 2012

State	Health Care, $ Million (Range)^a^	Renovation, $ Million (Range)^a^	Fires, $ Million (Range)^a^	Total, $ Million (Range)^a^
Alabama	3.09 (1.53–5.50)	2.78 (1.56–4.35)	0.65 (0.36–1.00)	6.52 (3.46–10.85)
Alaska	—b	—b	—b	—b
Arizona	0.81 (0.40–1.44)	0.17 (0.09–0.26)	0.14 (0.08–0.22)	1.11 (0.57–1.92)
Arkansas	1.05 (0.52–1.87)	1.30 (0.73–2.03)	0.21 (0.12–0.33)	2.56 (1.37–4.22)
California	7.80 (3.87–13.87)	0.50 (0.28–0.78)	0.69 (0.39–1.08)	8.99 (4.54–15.73)
Colorado	0.42 (0.21–0.76)	0.31 (0.17–0.48)	0.14 (0.08–0.22)	0.87 (0.46–1.45)
Connecticut	1.91 (0.95–3.41)	0.47 (0.26–0.73)	0.20 (0.11–0.31)	2.58 (1.33–4.45)
Delaware	0.27 (0.13–0.48)	0.08 (0.04–0.12)	0.03 (0.02–0.05)	0.38 (0.20–0.65)
District of Columbia	—b	—b	—b	—b
Florida	3.41 (1.69–6.06)	1.12 (0.63–1.74)	0.56 (0.32–0.88)	5.09 (2.64–8.69)
Georgia	2.54 (1.26–4.52)	1.35 (0.76–2.11)	0.64 (0.36–1.00)	4.53 (2.38–7.63)
Hawaii	0.95 (0.47–0.69)	0.21 (0.12–0.33)	0.11 (0.06–0.17)	1.28 (0.65–2.20)
Idaho	0.04 (0.02–0.07)	0.03 (0.01–0.04)	0.01 (<0.01–0.02)	0.08 (0.04–0.13)
Illinois	2.52 (1.25–4.49)	1.87 (1.05–2.92)	0.46 (0.26–0.72)	4.86 (2.57–8.14)
Indiana	1.00 (0.50–1.78)	1.04 (0.59–1.63)	0.24 (0.14–0.38)	2.29 (1.22–3.79)
Iowa	0.22 (0.11–0.39)	0.28 (0.16–0.44)	0.05 (0.03–0.08)	0.55 (0.30–0.91)
Kansas	0.40 (0.20–0.71)	0.67 (0.38–1.05)	0.12 (0.06–0.18)	1.18 (0.64–1.94)
Kentucky	1.19 (0.59–2.11)	1.76 (0.99–2.75)	0.28 (0.16–0.44)	3.23 (1.74–5.30)
Louisiana	2.47 (1.22–4.39)	1.01 (0.57–1.58)	0.35 (0.20–0.55)	3.83 (1.99–6.52)
Maine	0.80 (0.40–1.42)	0.22 (0.12–0.34)	0.07 (0.04–0.11)	1.09 (0.56–1.87)
Maryland	0.80 (0.40–1.43)	0.31 (0.17–0.48)	0.13 (0.07–0.20)	1.24 (0.64–2.11)
Massachusetts	5.90 (2.93–10.50)	1.52 (0.85–2.37)	0.49 (0.28–0.77)	7.91 (4.06–13.64)
Michigan	1.74 (0.86–3.10)	1.25 (0.70–1.95)	0.33 (0.18–0.51)	3.32 (1.75–5.56)
Minnesota	1.17 (0.85–2.09)	0.77 (0.44–1.21)	0.21 (0.12–0.33)	2.16 (1.14–3.63)
Mississippi	1.43 (0.71–2.54)	0.70 (0.39–1.09)	0.24 (0.14–0.38)	2.37 (1.24–4.01)
Missouri	1.14 (0.57–2.04)	1.23 (0.69–1.92)	0.27 (0.15–0.42)	2.64 (1.41–4.38)
Montana	0.12 (0.06–0.22)	0.12 (0.07–0.18)	0.04 (0.02–0.06)	0.28 (0.15–0.46)
Nebraska	0.36 (0.18–0.64)	0.40 (0.22–0.62)	0.10 (0.05–0.15)	0.85 (0.46–1.41)
Nevada	0.19 (0.09–0.34)	0.10 (0.06–0.16)	0.06 (0.04–0.10)	0.35 (0.19–0.60)
New Hampshire	0.28 (0.14–0.49)	0.21 (0.12–0.32)	0.06 (0.03–0.09)	0.54 (0.29–0.91)
New Jersey	2.59 (1.29–4.61)	1.63 (0.92–2.55)	0.52 (0.29–0.82)	4.75 (2.50–7.98)
New Mexico	0.58 (0.29–1.02)	0.11 (0.06–0.17)	0.08 (0.05–0.13)	0.77 (0.39–1.32)
New York	46.66 (23.17–83.01)	7.49 (4.21–11.70)	3.61 (2.03–5.65)	57.77 (29.41–100.36)
North Carolina	2.62 (1.30–4.67)	2.07 (1.17–3.24)	0.58 (0.32–0.90)	5.27 (2.79–8.81)
North Dakota	0.09 (0.04–0.16)	0.13 (0.07–0.20)	0.02 (0.01–0.04)	0.24 (0.13–0.39)
Ohio	3.26 (1.62–5.80)	2.53 (1.42–3.95)	0.69 (0.39–1.08)	6.48 (3.43–10.83)
Oklahoma	1.00 (0.50–1.78)	1.05 (0.59–1.64)	0.20 (0.11–0.31)	2.25 (1.20–3.73)
Oregon	0.29 (0.14–0.51)	0.11 (0.06–0.17)	0.05 (0.03–0.08)	0.45 (0.23–0.76)
Pennsylvania	3.62 (1.80–6.44)	2.91 (1.64–4.55)	0.64 (0.36–0.99)	7.17 (3.79–11.98)
Rhode Island	1.08 (0.54–1.92)	0.52 (0.29–0.81)	0.13 (0.07–0.20)	1.73 (0.90–2.93)
South Carolina	1.24 (0.62–2.21)	1.05 (0.59–1.64)	0.27 (0.15–0.42)	2.56 (1.36–4.28)
South Dakota	0.09 (0.04–0.16)	0.11 (0.06–0.17)	0.02 (0.01–0.03)	0.22 (0.12–0.36)
Tennessee	2.75 (1.37–4.89)	2.56 (1.44–4.00)	0.54 (0.31–0.85)	5.85 (3.11–9.74)
Texas	3.92 (1.95–6.97)	2.19 (1.23–3.43)	0.92 (0.52–1.43)	7.03 (3.70–11.83)
Utah	0.09 (0.04–0.15)	0.05 (0.03–0.08)	0.03 (0.02–0.05)	0.17 (0.09–0.28)
Vermont	0.29 (0.14–0.51)	0.07 (0.04–0.11)	0.02 (0.01–0.04)	0.38 (0.20–0.66)
Virginia	1.17 (0.58–2.09)	0.74 (0.42–1.16)	0.36 (0.20–0.56)	2.27 (1.20–3.81)
Washington	0.53 (0.26–0.94)	0.16 (0.09–0.24)	0.09 (0.05–0.14)	0.77 (0.40–1.32)
West Virginia	0.52 (0.26–0.93)	0.73 (0.41–1.15)	0.09 (0.05–0.15)	1.35 (0.72–2.22)
Wisconsin	1.02 (0.51–1.81)	0.58 (0.33–0.90)	0.17 (0.09–0.26)	1.76 (0.92–2.97)
Wyoming	0.05 (0.02–0.08)	0.07 (0.04–0.11)	0.01 (<0.01–0.02)	0.13 (0.07–0.22)
United States^c^	94.01 (46.67–167.24)	42.99 (24.18–67.17)	15.92 (8.95–24.87)	152.91 (79.81–259.28)

a Estimates by cost type may not equal total because of rounding.

b Estimates not presented due to lack of data on subsidized housing residency.

c Estimates exclude Alaska and the District of Columbia.

By state, total annual cost savings for public housing ranged from $0.08 million ($0.04–$0.13 million) in Idaho to $57.77 million ($29.41–$100.36 million) in New York (Table 2). By cost type, state annual cost savings for secondhand smoke-related health care ranged from $0.04 million ($0.02–$0.07 million) in Idaho to $46.66 million ($23.17–$83.01 million) in New York. State annual cost savings for renovation of smoking-permitted units ranged from $0.03 million ($0.01–$0.04 million) in Idaho to $7.49 million ($4.21–$11.70 million) in New York, and state annual cost savings for smoking-attributable fire losses ranged from $0.01 million (<$0.01–$0.02 million) in Idaho and Wyoming to $3.61 million ($2.03–$5.65 million) in New York.

## Discussion

This study reveals that prohibiting smoking in all US subsidized housing, including public housing, could yield annual cost savings of nearly a half billion dollars from averted secondhand smoke-related health care, renovation of units in which smoking has occurred, and smoking-attributable fire losses. By state, total annual cost savings could range from $580,000 in Wyoming to nearly $125 million in New York. These estimates include the cost savings that could be realized by prohibiting smoking in public housing alone, which would total approximately $153 million overall and range from $80,000 in Idaho to nearly $58 million in New York. These cost savings would be reaped by multiple stakeholders; for example, health care costs are primarily borne by taxpayers, employers, and state or federal government, whereas renovation and fire costs are generally borne by property owners or housing authorities and their insurance carriers. Nonetheless, irrespective of payer, efforts to prohibit smoking in all US subsidized housing, including public housing, would protect health and could be expected to generate substantial societal cost savings at the national and state levels. Implementing smoke-free policies in US market-rate multiunit housing would be expected to yield even greater societal cost savings and help protect the nation’s nearly 80 million multiunit housing residents from secondhand smoke exposure in their homes.

The findings from this study are generally consistent with those from the 2 published studies that estimated the economic cost savings achievable through the implementation of smoke-free multiunit housing policies. King and colleagues used a similar approach at the national level and reported estimated cost savings of approximately $521 million and $154 million annually for subsidized and public housing, respectively; the estimates in this study ($497 and $153, respectively) are lower because of adjustments for state variations in health care expenditures, Medicaid coverage, and smoke-free home rules (10). By cost type, national estimates for secondhand smoke-related health care and unit renovation were also comparable; however, estimated savings from smoking-attributable fires were lower in our study than those reported previously, because of recent declines in fire-attributable death costs reported by the National Fire Protection Association (19). Our state findings are also generally consistent with those of Ong and colleagues, who found that implementing smoke-free policies in all California multiunit housing would yield annual savings of $18 million from averted expenditures related to cleaning, repair, administration, and fire (11). The estimated savings from renovation and smoking-attributable fire losses for California in this study was $11 million, which is understandably lower considering inflation and the fact that multiunit subsidized housing comprises only a subset of the state’s multiunit housing.

Research shows that smoke-free policies are favored by most multiunit housing residents and are legally permissible in subsidized, public, and market-rate housing (21–23). The US Department of Housing and Urban Development has encouraged public housing authorities, and owners and managers of multifamily housing rental assistance programs such as Section 8, to implement smoke-free policies in their properties (24,25). As of October 2013, over 300 public housing authorities across the United States had instituted such policies, including all 20 in Maine (26). Given this study’s findings, it can be assumed that the implementation of these smoke-free policies in Maine public housing currently yield statewide cost savings of approximately $1.09 million per year. In addition to the public housing authorities that have implemented smoke-free policies across the country, at least 12 communities in California have enacted laws that prohibit smoking in all private units in market-rate multiunit housing and do not permit current residents to continue smoking in the prohibited areas (ie, no “grandfather clause”) (26). Additionally, a growing number of owners and managers of multiunit housing have voluntarily implemented such policies on their properties (27).

Despite existing evidence of the legal permissibility of smoke-free multiunit housing and strong support for such policies among residents, prevalence of such policies remains low. Additionally, many multiunit housing owners and managers have misconceptions about barriers to implementing such policies, including concerns about increased vacancy and turnover (21,27); however, the experiences of multiunit housing owners and managers who have implemented smoke-free policies suggest that these concerns are misplaced. For example, a cross-sectional survey of multiunit operators in Nebraska found that respondents without smoke-free policies expected vacancy (53.6%) and turnover (50.0%) rates to increase following policy implementation, whereas the proportion of operators with existing policies that reported experiencing these outcomes was 10.7% and 3.7%, respectively (21). This knowledge gap underscores the importance of educating multiunit housing owners and managers about the health and economic benefits of prohibiting smoking on their properties, including disseminating information on the experiences of their peers who have already successfully implemented such policies.

Concerns have been raised that smoke-free policies in subsidized housing could exacerbate socioeconomic disparities by adversely affecting low-income people and other vulnerable populations by displacing residents who refuse to comply (23). However, on balance, these policies actually have the potential to considerably reduce health disparities and the associated costs of secondhand smoke exposure across states, particularly considering the higher rates of secondhand smoke exposure among populations that traditionally comprise a large portion of subsidized housing, including children, racial/ethnic minorities, and residents of low socioeconomic status (5,9,28). This knowledge is particularly important in states such as New York, which would have noticeably higher cost savings than other states because approximately 15% of the country’s subsidized housing residents live in that state (9). It is also imperative to note that these policies prohibit the act of smoking, not the occupation of units by people who smoke. Moreover, research suggests that such policies do not lead to increased tenant turnover in subsidized housing and can help motivate smoking cessation and reduce cigarette consumption (29). Residents who quit smoking in response to smoke-free policies would probably experience improved health and realize cost savings through reduced use of health care services and tobacco purchases; tobacco purchases can comprise a substantial portion of low-income smokers’ income (30). These benefits could be maximized with the implementation of smoke-free multiunit housing policies in concert with the provision of evidence-based smoking cessation resources.

This study is subject to at least 5 limitations. First, all cost figures are based on estimates and assumptions, which are subject to uncertainty and variation. For example, some estimates were based on data from specific states, such as Minnesota and Maine, and may not be reflective of such costs in other states. However, state-specific data were used when available, adjustments were made to account for variations in the cost of living across states, and conservative estimates were used in all instances. Second, this analysis did not account for all societal costs associated with smoking. The inclusion of additional factors, such as long-term health care costs, indirect costs related to lost productivity from illness, or the benefits accrued by smokers who quit because of smoke-free policies, would yield higher estimates. The analysis also did not account for renovation costs associated with the infiltration of secondhand smoke into smoke-free units. Therefore, the findings are conservative and likely underestimates of actual cost savings. Third, health care cost estimates included only those medical conditions identified in the 2006 Surgeon General's report as having sufficient evidence of a causal link with secondhand smoke exposure (1). The current analysis did not include stroke, for which a causal designation was first noted in the 2014 Surgeon General’s report (2); thus, actual health care costs may be greater than those presented. Fourth, estimates were based on the number of residents whose housing is subsidized. Some residents of subsidized housing (eg, Section 8 housing) may live in the same building as residents whose housing is unsubsidized; the latter residents would also benefit from smoke-free policies, and including these residents in our analysis would probably yield higher cost savings. Finally, the analysis did not account for potential costs associated with policy implementation and enforcement, such as resident education, cessation support, and signage. However, research suggests that most multiunit housing operators who have implemented smoke-free policies report having no difficulty with policy enforcement; most use methods that require little investment of money or staff time, such as sending written warning letters (21). Many multiunit housing operators also report that the staff time devoted to managing buildings either stayed the same or decreased following policy implementation (21). Moreover, Medicaid cessation coverage is expanding, and free cessation support resources such as state telephone quitlines currently exist in all 50 states (3), thus reducing the need for substantial additional societal resources to support cessation among subsidized housing residents during smoke-free policy implementation.

This study indicates that prohibiting smoking in all US subsidized housing, including public housing, could result in annual savings of approximately $497 million, with state cost savings ranging from $580,000 in Wyoming to nearly $125 million in New York. These findings underscore the potential impact of smoke-free policies for protecting multiunit housing residents, visitors, and employees from this health hazard, as well as generating substantial societal cost savings at the national and state levels.
